# A Polyurethane Electrospun Membrane Loaded with Bismuth Lipophilic Nanoparticles (BisBAL NPs): Proliferation, Bactericidal, and Antitumor Properties, and Effects on MRSA and Human Breast Cancer Cells

**DOI:** 10.3390/jfb15100309

**Published:** 2024-10-16

**Authors:** Jesús Alejandro Torres-Betancourt, Rene Hernández-Delgadillo, Juan Valerio Cauich-Rodríguez, Diego Adrián Oliva-Rico, Juan Manuel Solis-Soto, Claudia María García-Cuellar, Yesennia Sánchez-Pérez, Nayely Pineda-Aguilar, Samantha Flores-Treviño, Irene Meester, Sergio Eduardo Nakagoshi-Cepeda, Katiushka Arevalo-Niño, María Argelia Akemi Nakagoshi-Cepeda, Claudio Cabral-Romero

**Affiliations:** 1 Laboratorio de Biología Molecular, Facultad de Odontología, Universidad Autónoma de Nuevo León, UANL, Monterrey 66455, Nuevo León, Mexico; jesus.torresbtn@uanl.edu.mx (J.A.T.-B.); rene.hernandezdl@uanl.edu.mx (R.H.-D.); uanlsolis@gmail.com (J.M.S.-S.); sergio.nakagoshicp@uanl.edu.mx (S.E.N.-C.); maria.nakagoshicp@uanl.edu.mx (M.A.A.N.-C.); 2 Instituto de Biotecnología, Facultad de Ciencias Biológicas, Universidad Autónoma de Nuevo León, UANL, Monterrey 66450, Nuevo León, Mexico; katiushka.arevalonn@uanl.edu.mx; 3 Centro de Investigación Científica de Yucatán, Unidad de Materiales, Mérida 97205, Yucatán, Mexico; juanvaleriocauich@hotmail.com; 4 Subdirección de Investigación Básica, Instituto Nacional de Cancerología, Ciudad de México 14080, Mexico; andor.diego@gmail.com (D.A.O.-R.); garcue57@gmail.com (C.M.G.-C.); s_yesennia@yahoo.com.mx (Y.S.-P.); 5 Centro de Investigación en Materiales Avanzados, S.C. (CIMAV), Subsede Monterrey, Apodaca 66628, Nuevo León, Mexico; nayely.pineda@cimav.edu.mx; 6 Servicio de Infectología, Hospital Universitario “Dr. José Eleuterio González”, Facultad de Medicina, Universidad Autónoma de Nuevo León, UANL, Monterrey 66455, Nuevo León, Mexico; samflorest@gmail.com; 7 Departamento de Ciencias Básicas, Escuela de Medicina, Universidad de Monterrey, San Pedro Garza García 66238, Nuevo León, Mexico; elisabethd.meester@udem.edu

**Keywords:** electrospun polyurethane membrane, *MRSA*, bismuth lipophilic nanoparticles, BisBAL NPs, tecoflex electrospun membrane, human breast cancer, antitumor activity, bactericidal activity, topical drug delivery system

## Abstract

Electrospun membranes (EMs) have a wide range of applications, including use as local delivery systems. In this study, we manufactured a polyurethane Tecoflex™ EM loaded with bismuth-based lipophilic nanoparticles (Tecoflex™ EMs-BisBAL NPs). The physicochemical and mechanical characteristics, along with the antitumor and bactericidal effects, were evaluated using a breast cancer cell line and methicillin-susceptible and resistant *Staphylococcus aureus* (MRSA). Drug-free Tecoflex™ EMs and Tecoflex™ EMs-BisBAL NPs had similar fiber diameters of 4.65 ± 1.42 µm and 3.95 ± 1.32 µm, respectively. Drug-free Tecoflex™ EMs did not negatively impact a human fibroblast culture, indicating that the vehicle is biocompatible. Tecoflex™ EMs-BisBAL NPs increased 94% more in size than drug-free Tecoflex™ EMs, indicating that the BisBAL NPs enhanced hydration capacity. Tecoflex™ EMs-BisBAL NPs were highly bactericidal against both methicillin-susceptible *S. aureus* and MRSA clinical isolates, inhibiting their growth by 93.11% and 61.70%, respectively. Additionally, Tecoflex™ EMs-BisBAL NPs decreased the viability of MCF-7 tumor cells by 86% after 24 h exposure and 70.1% within 15 min. Regarding the mechanism of action of Tecoflex™ EMs-BisBAL NPs, it appears to disrupt the tumor cell membrane. In conclusion, Tecoflex™ EMs-BisBAL NPs constitute an innovative low-cost drug delivery system for human breast cancer and postoperative wound infections.

## 1. Introduction

Breast cancer (BC) is the most prevalent malignancy among women worldwide, with approximately 1.38 million new cases each year. It is also the leading cause of cancer-related death among women, with an estimated 458,000 global fatalities annually [1]. In Latin America and the Caribbean, BC accounts for 27% of new cancer diagnoses and ranks fifth in cancer-related mortality. In Mexico, BC is the most prevalent type of cancer among women and the leading cause of death in women over 30 years old (INEGI; 2 February 2024), causing 11.7–17.0 deaths per 100,000 women annually [2].

Mastectomy is the preferred treatment for early-stage BC and generally has a good prognosis. However, mastectomy carries a risk of postoperative wound infections [3], particularly in immunocompromised patients with obesity, diabetes, and/or malnutrition [4]. Postoperative wound infections following surgery and chemotherapy extend recovery and hospitalization time [5], increasing the risk of additional nosocomial infections and resulting in extra healthcare costs. Surgical procedures disrupt skin and mucosal barriers that otherwise inhibit pathogen entry and colonization [6]. Among the pathogens isolated from post-mastectomy wound infections are *Staphylococcus aureus* and *Streptococcus pyogenes* [7]. The endotoxins synthesized by *S. aureus* (toxin-1; TSST-1) can act as potent superantigens that activate the immune system excessively and are a cause of the toxic shock syndrome [8].

The possibility of BC recurrence after surgical removal is high, especially when long-term monitoring is considered. Factors such as a positive lymph node (pN status), large tumor size, and estrogen receptor-positive tumors increase the risk of recurrence. Late recurrence of up to 32 years after primary diagnosis has been reported [9]. In Mexico, pN status and a positive response to chemotherapy are key factors in BC recurrence. These patients often receive radiation and endocrine therapy to combat recurrence. However, the undesirable side effects constitute a huge disadvantage and can lead to discontinuation of the treatment. A biomedical device with good biocompatibility and antitumor properties applied at the tumor surface could interfere with tumor growth and reduce the likelihood of BC recurrence [10].

Unfortunately, there are no biomaterials with both antimicrobial and antitumor properties for use as topical treatments after surgery in BC patients. Such biomaterials would reduce the risk of BC recurrence and postoperative wound infections. Electrospinning is a novel technique that uses high voltages to create micro- and nanofiber polymeric scaffolds from natural and synthetic polymers [11,12]. Electrospun membranes (EMs) can mimic the structure of the extracellular matrix and serve as cellular scaffolds with the potential to integrate with adjacent tissues. Therefore, EMs are widely used in tissue engineering applications [13,14]. Among the most popular EM types for tissue engineering are polyvinyl alcohol (PVA) and polyurethanes, especially Tecoflex^TM^. Polyurethane elastomers are copolymers made of hard and soft segments. Hard chain extenders are composed of aliphatic diisocyanates (ADIs; intermediate chemicals employed to develop coatings, adhesives and textiles), while flexible and elastic segments are made of diol-terminated polyethers, polybutadienes, or polyesters [12].

Tecoflex^TM^ adheres to and is biocompatible with human fibroblasts [15]. Because of their mechanical and biocompatibility characteristics, polyurethane polymers have been applied in biomedical applications for several decades [16]. To our knowledge, there are no reports on Tecoflex^TM^ as a delivery tool for antimicrobial or antitumoral drugs, but there are reports for other EM types. A PVA-based EM loaded with silver nanoparticles was studied for the growth inhibition of *S. aureus* and *Escherichia coli* [17]. A plasma-modified, electro-spun 3D scaffold has been used in the human cancer microenvironment for individualized therapeutic screening [18]. Another polyurethane (PCL-Diol-b-PU) loaded with the antitumoral agent temozolomide has been tested for treating glioblastoma [19].

Previously, we reported on the selective but potent antimicrobial and antitumor properties of bismuth lipophilic nanoparticles (BisBAL NPs) [20,21]. Our group characterized the antimicrobial and antibiofilm properties of BisBAL NPs via their effects on protozoans, fungi, and bacteria. BisBAL NPs inhibited the growth of both Gram-positive and Gram-negative bacteria, including multi-resistant microorganisms like methicillin-resistant *Staphylococcus aureus* (MRSA), and diminished MRSA biofilm formation on human bone surfaces [20,22]. BisBAL NPs have been used to supplement various materials, such as root canal cement sealer (AH Plus), conferring antimicrobial and antibiofilm properties [23]. The selective antitumor effect of BisBAL NPs against MCF-7 human BC cells was dose-dependent; 25 µM BisBAL NPs inhibited MCF-7 growth by 80.4% [21]. The action mechanisms of BisBAL NPs on tumor cells include loss of plasma membrane integrity and genotoxicity [19]. BisBAL NPs are not cytotoxic to healthy cells and tissues in in vitro and in vivo models, suggesting a potential for clinical application [24]. In general, bismuth-based nanomaterials (Bi-based NM) have shown minimal cytotoxicity to healthy human cells following biodistribution and renal excretion, demonstrating their potential clinical usefulness in a range of medical applications [25].

This study investigated a topical delivery tool for BisBAL NPs as a next step toward its clinical application. We loaded commercially available medical-grade polyurethane Tecoflex™ with BisBAL NPs and evaluated its bactericidal and antitumor properties. Also, the effect of Tecoflex^TM^ on human fibroblast proliferation was studied. Tecoflex™ could be used on postoperative wounds as a topical delivery agent for BisBAL NPs to prevent both microbial infection and BC regrowth, without adverse side effects.

## 2. Materials and Methods

### 2.1. Synthesis and Characterization of BisBAL NPs

BisBAL NPs were synthesized using the colloidal method [20]. All chemicals were of analytical grade and acquired from Sigma-Aldrich (St. Louis, MO, USA). Briefly, bismuth nitrate was reduced with NaBH4, 0.45 g (Bi(NO_3_)_3_·5H_2_O was dissolved in 20 mL of propylene glycol, requiring 2 h of heating at 80 °C with occasional vortexing. The (Bi(NO_3_)_3_·5H_2_O solution was mixed with 2,3-dimercapto-1-propanol in a 2:1 molar ratio to generate BisBAL NPs. The BisBAL NPs were characterized by scanning electron microscopy (SEM; Carl Zeiss Auriga FIB-SEM & TEM, Oberkochen, Germany) to determine their form, size, and arrangement. Energy-dispersive X-ray spectroscopy (EDX; Oxford INCA X-Sight, Tubney Woods, UK) was employed for chemical composition analysis. X-ray diffraction patterns were obtained using an X-ray diffractometer (Panalytical X’Pert PRO MRD) equipped with Cu Kα as an X-ray source (λ = 1.541874 Å).

### 2.2. Fabrication of Tecoflex™ EMs and Coating with BisBAL NPs

An 8% stock solution (*w*/*v*) of Tecoflex™ SG-80A (Lubrizol Advanced Materials; EC, Cleveland, OH, USA) in chloroform (MERCK; Darmstadt, Germany) was prepared by dissolving 0.8 g of Tecoflex™ SG-80A in 10 mL of chloroform at 25 °C until homogeneous. BisBAL NPs were added to achieve a final concentration of 1 mM. To create the EMs, a 20-mL plastic syringe, equipped with a steel needle (internal diameter, 800 µm) was filled with either drug-free or Tecoflex™ EMs-BisBAL NPs. Electrospinning was performed using a NEU-01 Standard Unit (Tong Li Tech Co, Nanshan, Shenzhen, China) at room temperature. For drug-free Tecoflex™ EM, the parameters were as follows: +22 kV voltage at the needle, −22 kV voltage at the collector, feed rate of 0.5 mL/h, and distance of 18 cm between injector and collector. For Tecoflex™ EMs-BisBAL NPs, 12 kV was applied to the positive electrode, and a vertical flat collector was used. EMs were coated for 30 min (final concentration of BisBAL NPs, 10 mM) at room temperature. The Tecoflex™ EMs containing BisBAL NPs (Tecoflex™ EMs-BisBAL NPs) were cut into circles (diameter, 6 mm) for characterization.

### 2.3. Physicochemical Characterization of Tecoflex™ EMs-BisBAL NPs (SEM, FTIR, and Raman)

Optical and scanning electron microscopy (SEM; FEI Tecnai G2 Twin, Hillsboro, OR, USA; 160 kV accelerating voltage) were used to analyze the Tecoflex™ EMs-BisBAL NPs and drug-free Tecoflex™ EMs. The elemental composition was studied using energy-dispersive X-ray spectroscopy (EDS; Oxford INCA X-Sight, Tubney Woods, UK).

Fourier-transform infrared (FTIR) spectra of both Tecoflex™ EMs-BisBAL NPs and drug-free Tecoflex™ EMs were obtained using attenuated total reflectance (ATR) with ZnSe crystals and a Nicolet 8700 FTIR spectrophotometer (Thermo Scientific, Madison, WI, USA). Scans were acquired over a spectral range of 4000–650 cm^−1^, averaging 200 scans with a resolution of 4 cm^−1^. Raman spectra (range, 3200 cm^−1^–100 cm^−1^; power, 100%) were acquired using a Renishaw InVia spectrometer (Reinshaw Gloucestershire, UK) equipped with a 633/532 nm laser set at a 10 s exposure.

### 2.4. Water Absorption Capacity of Tecoflex™ EMs-BisBAL NPs

To assess the water-retention properties of Tecoflex™ EMs-BisBAL NPs, 1-cm^2^ squares (n = 3) were incubated in 10 mM phosphate buffered saline (PBS) for 24 h at 37 °C. The EM squares were weighed before (dry weight; Wd) and after (wet weight; Ww) with an analytical balance. The retention percentage (RR) was calculated as follows: RR = ((Ww − Wd)/Wd) × 100.

### 2.5. Tecoflex™ EM-BisBAL NPs Tensile Test

The tensile mechanical properties of Tecoflex™ EM BisBAL NPs and drug-free Tecoflex™ EM were analyzed using a Shimadzu AGS-X testing machine (Kyoto, Japan). Rectangular samples (25 mm × 5 mm) of Tecoflex™ EM-BisBAL NPs were cut with sterile surgical scissors. The tensile test was conducted using a 10 N cell with a cross-head speed of 10 mm/min at 25 °C. At least three samples were measured for each EM.

### 2.6. Biological Properties of Tecoflex™ EMs-BisBAL NPs

#### 2.6.1. Cell Culture

The human BC cell line MCF-7 (ATCC, HTB-22; Rockville, MD, USA) and human gingival fibroblasts (HGFs; non-tumor controls; kindly donated by Dr. Higinio Arzate: Dentistry faculty, UNAM) were cultured in DMEM/Ham’s F12 medium (DMEM/F12) supplemented with 15% fetal bovine serum (FBS; Gibco-invitrogen, Carlsband, CA, USA) and antibiotics (100 U/mL penicillin, 100 µg/mL streptomycin, and 0.25 µg/mL amphotericin B; Sigma-Aldrich Corporation, St Louis, MO, USA) in culture flasks (Corning Inc., Corning, NY, USA) at 37 °C in a humidified atmosphere with 5% CO_2_.

#### 2.6.2. Proliferation Assay

Proliferation experiments were conducted in 96-well plates containing sterile, drug-free Tecoflex^TM^ EM. HGFs (1 × 10^5^) were seeded onto the EMs and incubated at 37 °C in a 5% CO_2_ atmosphere for 24, 48, and 72 h. HGF monolayers were detached from the Tecoflex^TM^ EM with 0.005% trypsin, and the cell suspension was centrifuged (900 rpm, 5 min). Cell pellets were resuspended in PBS, and cell counts were performed in triplicate using a hemocytometer. Cells were stained with SYTO^TM^ 9 (10 µM as final concentration) (Thermo Fisher Scientific, Carlsbad, CA, USA) using the FITC filter. The cell morphology of green-stained cells confirmed cell viability.

#### 2.6.3. MRSA Isolation and Identification

A clinical isolate of MRSA was obtained from the Laboratory of Infectious Diseases at the University Hospital “Dr. José Eleuterio González”, a tertiary-care teaching hospital in Monterrey, Mexico. Species identification was performed using matrix-assisted laser desorption/ionization time-of-flight mass spectrometry (MALDI-TOF MS, Microflex LT system, Bruker Daltonics, Bremen, Germany) according to the manufacturer’s instructions. Single colonies from a 24 h culture on blood agar plates (37 °C) were placed on a 96-spot stainless steel target plate (Bruker Daltonics), and 1 µL of 70% formic acid and 1 µL of alpha-cyano-4-hydroxycinnamic acid matrix solution (Sigma-Aldrich, Toluca, Mexico) were added. The plate was analyzed with Biotyper 3.0 software (Bruker Daltonics) to identify matches with the spectral profile database using the following criteria: genus score, 2.000–2.299; species score, 2.300–3.000. To obtain high-quality images, spectra were captured using flexControl V.3.4 software (Bruker Daltonics), and a smoothing process was carried out with the Savitzky/Golay algorithm (10 cycles; width, 2 m/z). The Top Hat algorithm was used for baseline subtraction.

#### 2.6.4. Antimicrobial Activity against MRSA Clinical Isolate

Antimicrobial susceptibility was determined by broth microdilution using the VITEK 2.0 (bioMérieux, Marcy-l’Étoile, France) according to the manufacturer’s instruction. Bacteria were incubated on trypticase soy agar plates at 35 °C overnight. Next, a bacterial suspension (McFarland, 0.5/0.45% NaCl) was introduced into the equipment, and AST-GP75 VITEK cards for Gram-positive species were inoculated. After overnight incubation at 35 °C, the minimum inhibitory concentration (MIC) was obtained, and isolates were classified as susceptible, intermediate, or resistant based on current Clinical and Laboratory Standards Institute (CLSI) breakpoints criteria (CLSI, 2023).

#### 2.6.5. Bacterial Culture

Methicillin-susceptible *S. aureus* (MSSA-ATCC 29213; Rockville, MD, USA) and the *MRSA* clinical strain (*MRSA*-CI) were grown in trypticase soy broth agar (TSB; BD DIFCO, Sparks, MD, USA) at 37 °C for 24 h under aerobic conditions.

#### 2.6.6. Bactericidal Activity of Tecoflex™ EMs-BisBAL NPs

The bactericidal potential of Tecoflex™ EMs-BisBAL NPs was analyzed according to Tian et al., 2013, who described a procedure for similar EM supplementation with silver nanoparticles [26]. Disk diffusion assays were conducted with MSSA-ATCC and MRSA-CI. Standard inocula (100 µL, 0.5 McFarland) were plated on TSB agar Petri dishes, followed by the addition of Tecoflex™ EMs-BisBAL NPs, drug-free Tecoflex™ EM (negative control of growth inhibition), 10 mM vancomycin (positive control of growth inhibition) and 10 mM BisBAL NPs (n = 3). For quantitative analysis, serial dilutions and colony counting assays [27] were performed. MSSA-ATCC and MRSA-CI strains were exposed for 3 h (37 °C, aerobic conditions) to Tecoflex™ EMs-BisBAL NPs, drug-free Tecoflex™ EM, or filter paper with 10 mM vancomycin. After incubation, the third dilution was plated and incubated for 24 h at 37 °C under aerobic conditions, and the colony-forming units (CFUs) were counted. Results from triplicates were determined as percentages and compared among groups.

#### 2.6.7. Antitumor Activity of Tecoflex™ EMs-BisBAL NPs

The antitumor activity of Tecoflex™ EMs-BisBAL NPs on MCF-7 cells was analyzed with the PrestoBlue cell viability assay (Life Technologies Corporation, Carlsbad, CA, USA) [28]. MCF-7 cells were plated in 96-well cell culture plates at a density of 1 × 10^5^ cells/mL and incubated (37 °C; 5% CO_2_; 24 h). Cells were then exposed to Tecoflex™ EMs-BisBAL NPs, drug-free Tecoflex™ EMs (negative control), and 1 mM doxorubicin (DOX; positive control of growth inhibition) for 24 h (37 °C; 5% CO_2_). Following exposure, PrestoBlue reagent was added (10 µL/well, 30 min, 37 °C, 5% CO_2_) according to the manufacturer’s instructions. Absorbance at 570 nm was measured using a 96-well plate reader (Biotek, Winooski, VT, USA). Cell viability was expressed as percentage of the growth control.

#### 2.6.8. Action Mechanism of Tecoflex™ EMs-BisBAL NPs

BisBAL NPs are known to damage and alter the cell membrane permeability of tumor cells [21]. To verify whether the BisBAL NPs incorporated within Tecoflex™ EMs conserved this action mechanism, calcein AM location was monitored by fluorescence microscopy. Briefly, MCF-7 cells were exposed for 24 h (37 °C; 5% of CO_2_) to EMs-BisBAL NPs, drug-free Tecoflex™ EM (negative control), or 1 mM DOX (growth inhibition control). After washing with cold PBS, cells were incubated for 30 min (37 °C; 5% CO_2_) with 2 µM calcein AM (Biotium, Fremont, CA, USA). Non-internalized dye was washed out with PBS, and cells were dried in the dark before observation with an EVOS Cell Imaging System (Thermo Fisher Scientific, Carlsbad, CA, USA) using the FITC filter.

### 2.7. Statistical Analysis

One-way analyses of variance (ANOVAs) were employed to compare parametric data among the groups. A significance level of α = 0.05 was considered.

## 3. Results

### 3.1. Characterization of BisBAL NPs

**Figure 1:**
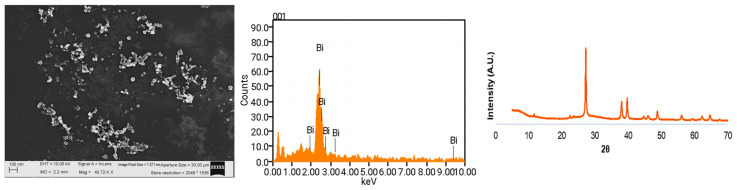
Characterization of BisBAL NPs by scanning electron microscopy (SEM). BisBAL NPs morphology, size, and distribution were obtained by SEM (SEM; Carl Zeiss Auriga FIB-SEM & TEM, Oberkochen, Germany). The chemical composition and X-ray diffraction pattern corroborated bismuth (Bi) presence within the samples.

The BisBAL NPs synthesized by the colloidal method were spheres with an average diameter of 28 nm (Figure 1) that formed agglomerates, which is a common observation for metallic nanoparticles. The EDX spectrum and X-ray diffraction pattern were indicative of the specific presence of bismuth in the nanostructures (Figure 1). Altogether, these data prove that the BisBAL NPs had the desired chemical composition, free from contamination.

### 3.2. Scanning Electron Microscopy of Tecoflex™ EMs-BisBAL NPs

**Figure 2:**
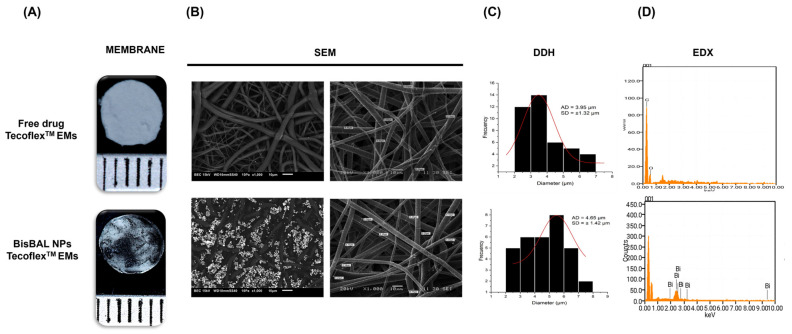
Characterization of Tecoflex™ EMs-BisBAL NPs by SEM-EDX. (**A**) Physical appearance of drug-free and Tecoflex™ EMs-BisBAL NPs. (**B**,**C**) SEM revealed similar fiber size. Drug-free Tecoflex™ EM is white, whereas Tecoflex™ EMs-BisBAL NPs has a grey hue. (**D**) EDX spectra were similar except for the bismuth peaks.

Uniformly white Tecoflex™ EMs featured a mottled-marbled appearance with black patches and streaks after the incorporation of BisBAL NPs (Figure 2A). Electrospun polyurethane membranes (Tecoflex™ SG-80A) with or without BisBAL NPs were observed via SEM (Figure 2B). Drug-free Tecoflex™ EMs resembled white filter paper made up of fibers with an average diameter of 3.95 ± 1.32 µm, while Tecoflex™ EMs-BisBAL NPs had a black hue over the entire surface and the average fiber diameter was 4.65 ± 1.42 µm (Figure 2B,C). BisBAL NPs within Tecoflex™ EMs-BisBAL NPs accumulated mainly along the surface of the fibers (Figure 2B). The chemical analysis by EDX spectra revealed similar profiles for the drug-free and Tecoflex™ EMs-BisBAL NPs for basic elements such as carbon (C) and oxygen (O); however, bismuth (Bi) was detected only in the Tecoflex™ EMs-BisBAL NPs (Figure 2D).

### 3.3. FTIR, Raman, Swelling and Tensile Strength Characterization of Tecoflex™ EMs-BisBAL NPs

**Figure 3:**
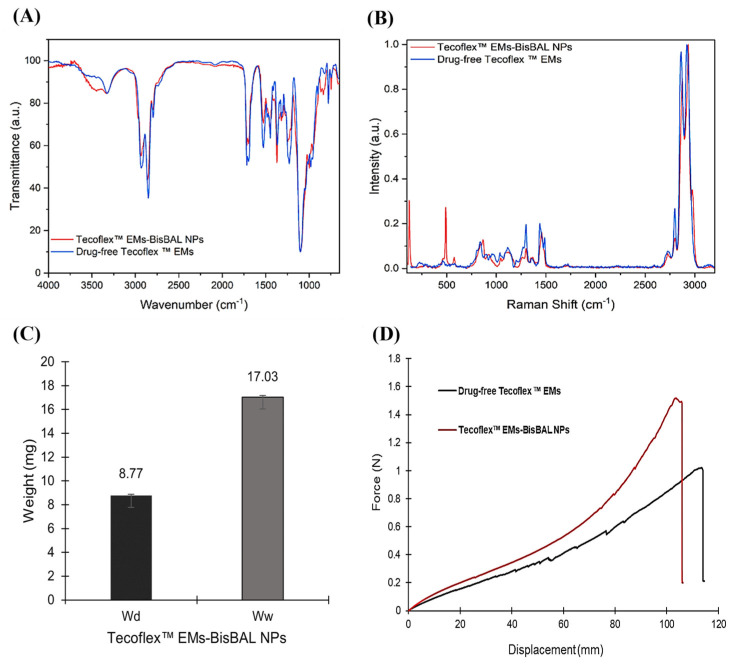
FTIR, Raman, water absorption, and mechanical characteristics of Tecoflex™ EMs–BisBAL NPs. (**A**) Raman spectra, (**B**) FTIR spectra, and (**C**) Swelling pattern of Tecoflex–EMs-BisBAL NPs and drug-free Tecoflex™ EMs. Bars indicate mean ± SD, n = 3. (**D**) The load–displacement curve of Tecoflex™ EMs BisBAL NPs and drug-free Tecoflex™ EM.

The FITR chemical composition spectra (Figure 3A) are summarized in Table 1. The main differences between the drug-free (blue) and BisBAL-NP (red)-containing Tecoflex™ EMs (Figure 3A) were (i) the NH region split in the main absorptions located at 3449 and 3334 cm^−1^, (ii) a small shift in the CH_2_ bending, (iii) a displacement of the C-O-C, and (iv) a few new bands between 1000 and 935 cm^−1^.

Likewise, the Raman spectra of drug-free Tecoflex™ and Tecoflex™ EMs-BisBAL NPs were quite similar (Figure 3B), with shared peaks at (i) 2921, 2862, and 2798 cm^−1^ corresponding to CH absorptions; (ii) low-intensity peaks at 1720 cm^−1^ assigned to C=O; (iii) CH2 absorption peaks at 1486 and 1438; and (iv) absorption peaks at 1298, 1115, and 834 cm^−1^. Peaks at 1037, 966, and 900 cm^−1^ displayed some shifts in line with the FTIR spectra. The incorporation of BisBAL NPs did not generate major differences in the Raman spectrum compared with the drug-free version, probably due to their low concentration. Tecoflex™ EM-BisBAL NPs displayed excellent water retention behavior (94%) after 24 h submersion in PBS (Figure 3C). With respect to mechanical characteristics, the load–displacement curves (Figure 3D) revealed a tensile strength of 1.56 N ± 0.84 SD for Tecoflex™ EM BisBAL NPs and 0.95 N ± 0.23 SD for drug-free Tecoflex™ EM.

### 3.4. Proliferation Assay

**Figure 4:**
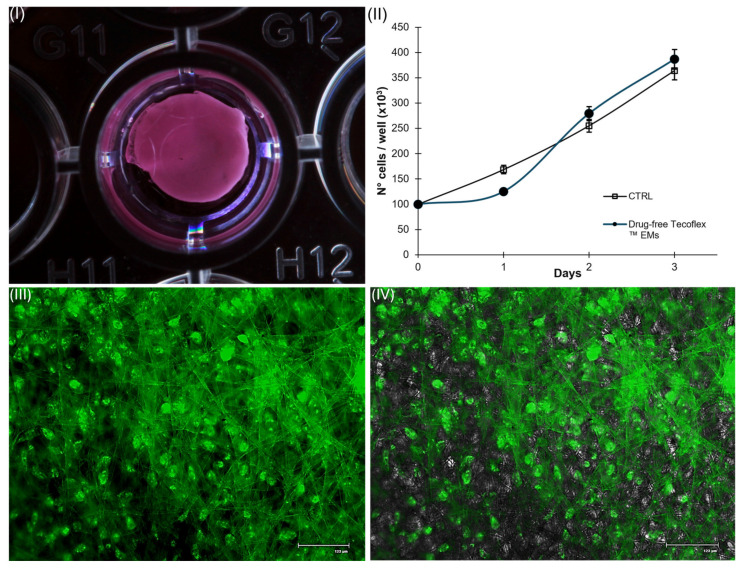
Proliferation assay of drug-free Tecoflex™ EMs on HGFs. A HGF culture on drug-free Tecoflex™ EMs was analyzed after 24, 48, and 72 h post-incubation (37 °C; 5% CO_2_). Cells grown without EMs served as a growth control (CTRL). (**I**) Cells seeded on top of drug-free Tecoflex™ EMs in a 96-well plate. (**II**) HGF´s growth curves in time when exposed to drug-free Tecoflex™ EMs for 24, 48, and 72 h or not. Bars indicate mean ± SD, n = 3. At day 3, there was no statistically significant growth difference between HGFs cultured on drug-free Tecoflex™ EMs and controls. (**III**) HGFs culture on drug-free Tecoflex™ EMs were observed by fluorescent microscopy after SYTO^TM^ 9 staining. (**IV**) Merging of SYTO^TM^ 9 HGFs (fluorescence microscopy) and drug-free Tecoflex™ EMs fibers (clear field microscopy).

Both control cells and HGFs cultured on drug-free Tecoflex™ EM (Figure 4I) multiplied their cell numbers almost four-fold in three days, with no statistically significant difference between the groups. However, where the growth rate of the control cells was linear, HGFs on drug-free Tecoflex™ EM displayed a 1-day growth delay, followed by exponential growth on day 2 that surpassed the control´s growth rate, and this advantage was maintained through day 3 (Figure 4II). Cells cultured on drug-free Tecoflex™ EM showed their typical morphology after SYTO^TM^ 9 staining (Figure 4III). A merged image suggests that HGFs cells adhered to the drug-free Tecoflex™ EMs fibers (Figure 4IV). Altogether, these results support the hypothesis that drug-free Tecoflex™ EMs promote HGFs proliferation.

### 3.5. Identification and Resistance Analysis of a Bacterial Clinical Isolate

**Figure 5:**
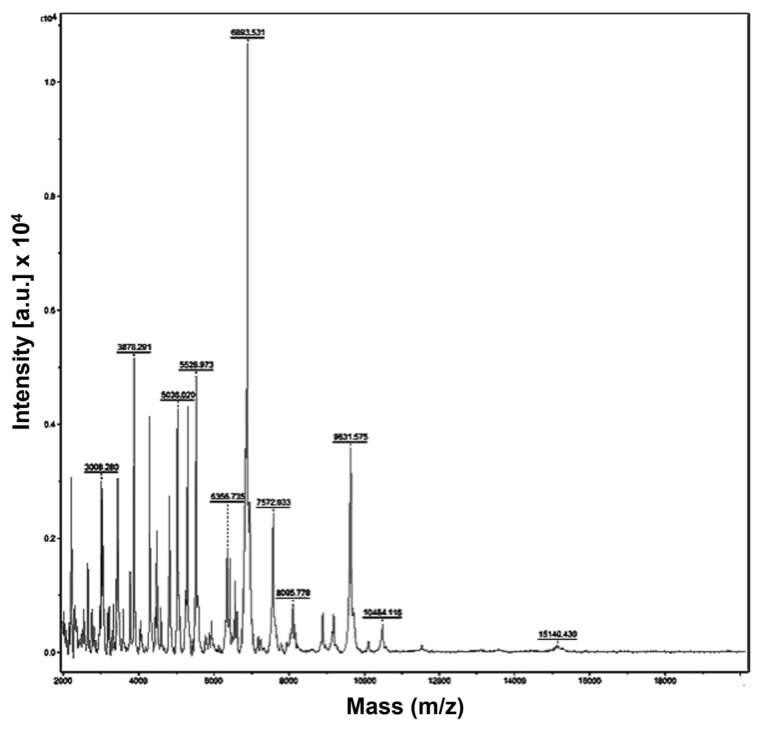
MALDI-TOF mass spectra of *MRSA*-CI. Software, flexAnalysis (Bruker Daltonics): *MRSA* spectrum.

The MRSA-CI strain was isolated from a breast lesion from a female patient from the Internal Medicine ward during 2023 and was identified as *S. aureus* isolate 23–2672 (Figure 5). AST data revealed resistance to oxacillin (i.e., methicillin-resistance) and susceptibility to ciprofloxacin, levofloxacin, moxifloxacin, clindamycin, daptomycin, erythromycin, gentamycin, tetracycline, trimethoprim, and linezolid.

### 3.6. Bactericidal Activity of Tecoflex™ EMs-BisBAL NPs

**Figure 6:**
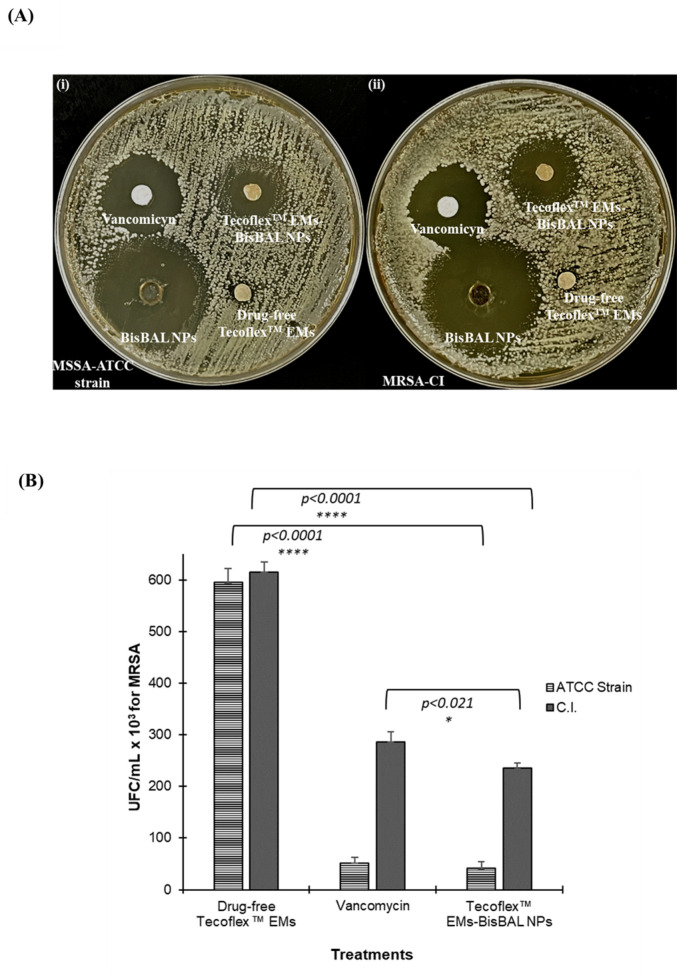
Bactericidal activity of Tecoflex™ EMs-BisBAL NPs against methicillin-susceptible and resistant *S. aureus* (*MSSA*-ATCC and *MRSA*-CI, respectively). (**A**) Disk diffusion assay after 24 h exposure to Tecoflex™ EMs-BisBAL NPs, drug-free Tecoflex™ EM (negative control), 10 mM vancomycin (inhibition control), and 10 mM BisBAL NPs. (i) MSSA-ATCC strain and (ii) MRSA-CI. (**B**) Colony counting assay. Bacterial suspensions were serially diluted and exposed to the indicated drugs for 3 h. Next, the bacteria were plated, and the UFCs were counted after 24 h. Bactericidal activity was significantly increased with Tecoflex^TM^ EMs-BisBAL NPs treatment compared with vancomycin for MRSA C.I. (* *p* < 0.021). Bars indicate mean ± SD (n = 4); asterisks indicate statistical significance.

Disk diffusion assays showed that, unlike drug-free Tecoflex™ EM, Tecoflex™ EMs-BisBAL NPs, were highly bactericidal against both MSSA-ATCC and the clinical isolate MRSA-CI (Figure 6A). Tecoflex™ EM-BisBAL NPs had a bactericidal capacity comparable to vancomycin, which is used in clinical settings against MRSA. Interestingly, the bactericidal activity of BisBAL NPs was higher than that of vancomycin against both MSSA-ATCC and MRSA-CI. The latter is relevant because MRSA is considered one of most dangerous pathogens in nosocomial infections. With the aim of obtaining quantitative data, serial dilution and a colony counting assay were used. Vancomycin (10 mM) reduced growth of MSSA by 91.32% and MRSA growth by 53.41%, while Tecoflex™ EMs-BisBAL NPs had stronger bactericidal effects, reducing growth by 93.11% and 61.70%, respectively (Figure 6B). Drug-free Tecoflex™ EMs did not inhibit bacterial growth for either *S. aureus* strain (Figure 6B). Altogether, these results reveal the strong and fast bactericidal capacity of Tecoflex™ EMs-BisBAL NPs, with an efficacy at least as good as vancomycin, which is currently one of the best antibiotics against MRSA infections.

### 3.7. Antitumor Potential of Tecoflex™ EMs-BisBAL NPs

**Figure 7:**
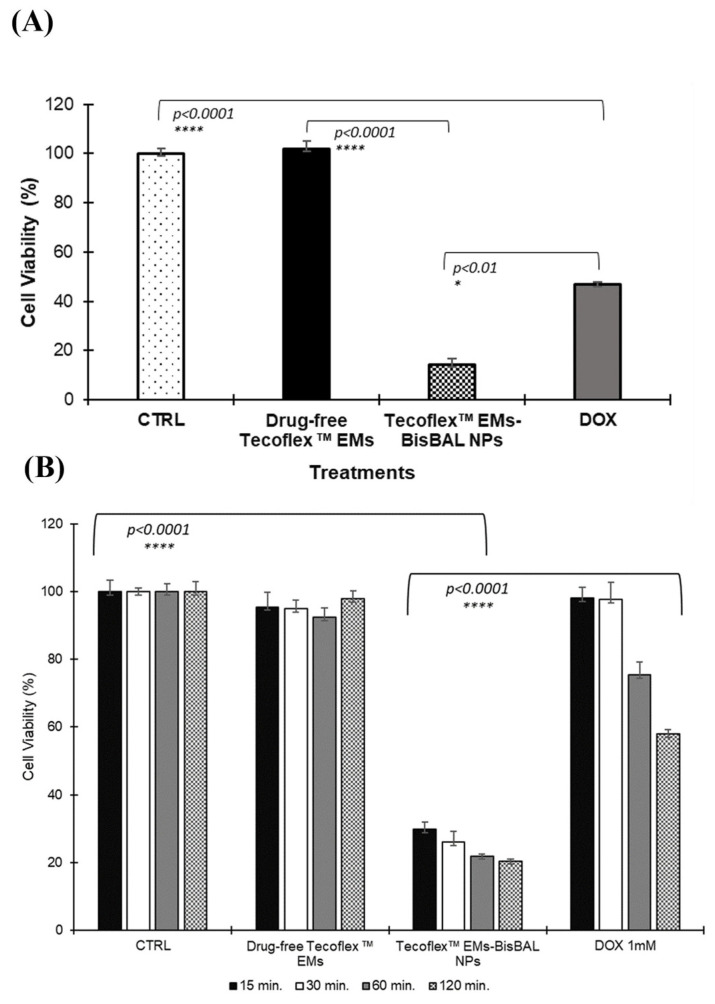
Antitumor activity of Tecoflex™ EMs-BisBAL NPs on human breast cancer cells. (**A**) Presto Blue cell viability assay was used to measure the ability of Tecoflex™ EMs-BisBAL NPs to inhibit the proliferation of MCF-7 cells. Drug-free Tecoflex™ EMs were used as negative control and 1 mM doxorubicin (DOX) as growth inhibition control. After 24 h incubation (37 °C; 5% CO_2_), cell viability was measured and expressed as percentage of a non-exposed growth control (CTRL). (**B**) The efficiency of the drugs was verified by repeating the experiment with shorter exposure times (15, 30, 60, and 120 min). Antitumoral activity was significantly increased with Tecoflex^TM^ EMs-BisBAL NPs treatment compared with growth control and drug-free Tecoflex™ EM (*p* < 0.0001), except for doxorubicin group with a significance difference of *p* < 0.01. Bars indicate mean ± SD (n = 3); asterisks indicate statistical significance.

When the antitumor activity was analyzed against the human breast tumor cell line MCF-7, Tecoflex™ EMs-BisBAL NPs caused an 86% growth reduction (Figure 7A), outperforming the inhibition control, 1 mM DOX, which caused a 53% growth reduction. Drug-free Tecoflex™ EMs did not interfere with MCF-7 growth (Figure 7A). Interestingly, a 15 min exposure to Tecoflex™ EMs-BisBAL NPs sufficed to reduce MCR-7 growth by 70.1%, while 1 mM DOX reduced MCF-7 growth only by 2% in this time span (Figure 7B). Thus, Tecoflex™ EMs-BisBAL NPs seems more efficient than 1 mM DOX against MCF-7 in vitro.

### 3.8. Action Mechanism of Tecoflex™ EMs-BisBAL NPs

**Figure 8:**
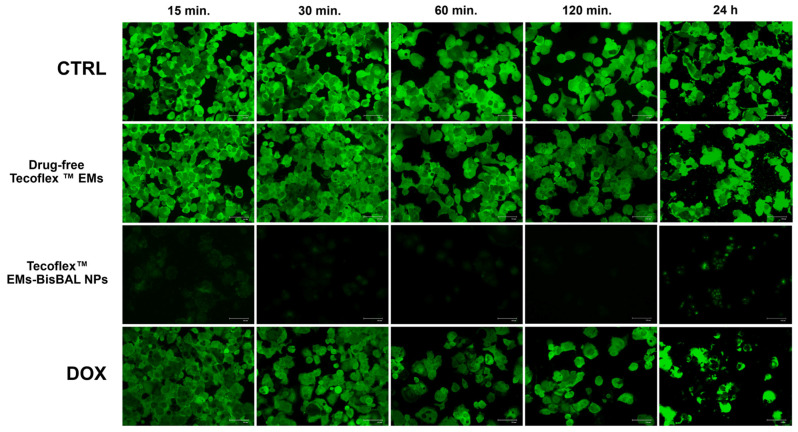
The effect of Tecoflex™ EMs-BisBAL NPs on MCF-7 viability and membrane permeability. MCF-7 cells were exposed for 15 min–24 h to Tecoflex™ EMs-BisBAL NPs and controls, before verifying cell viability and membrane permeability by calcein AM exposure according to the manufacturer´s instruction. Tumor cells grown in culture media without exposure to the drug were used as a growth control (CTRL), while 1 mM doxorubicin (DOX) served as a growth inhibition control.

To verify the importance of membrane permeability for the anti-tumor activity of Tecoflex™ EMs-BisBAL NPs, calcein AM was monitored by fluorescence microscopy. After 24 h exposure to Tecoflex™ EMs-BisBAL NPs or drug-free Tecoflex™ EMs, MCF-7 cells were exposed to membrane-permeable calcein AM. Once internalized, calcein can become fluorescent after hydrolyzation by cytoplasmic esterases. Green-fluorescent cells indicate healthy cells with an intact membrane, as can be observed with the growth control (CTRL, Figure 8), the exposure to drug-free Tecoflex™ EM, and the short-term (<30 min.) exposure to 1 mM DOX. Longer exposures to DOX caused cell death. Importantly, MCF-7 cells exposed to Tecoflex™ EMs-BisBAL NPs abolished tumor cell viability following 15 min exposure (Figure 8). The presence of a few green dots at 24 h may indicate that the dye was internalized and metabolized, suggesting that membrane damage had occured.

## 4. Discussion

Despite technological progress in early cancer detection and treatments, BC remains a significant challenge for modern medicine. Worldwide, BC has the highest incidence and mortality rates among women over 30 years old [29]. Currently, early detection and effective surgery are crucial for successful recovery. However, despite modern surgical methods and laser techniques, BC is a recurring event in a significant proportion of patients [30,31]. Recurrent BC is relatively frequent among BC patients who received adjuvant endocrine treatment [32,33]. Unfortunately, there is no topical treatment available for recurrent BC.

In this study, we developed and characterized an innovative EM loaded with BisBAL NPs as the active substance and evaluated its antitumor and bactericidal properties. The Tecoflex™ EMs-BisBAL NPs comprised a scaffold of fibers with an average diameter of 4.74 µm, with BisBAL NPs distributed homogenously on the surface of the fibers. A similar composition has been reported for silver nanoparticles on fabricated electrospun nanofibers [34]. The average fiber diameter of drug-free Tecoflex™ EMs was smaller (3.97 µm) compared with the Tecoflex™ EMs-BisBAL NPs (4.74 µm), probably due to the experimental manufacturing conditions. The difference in fiber diameters between drug-free Tecoflex™ EMs and Tecoflex™ EMs-BisBAL NPs is reflected in their specific mechanical properties. Compared with the drug-free Tecoflex™ EMs, Tecoflex™ EMs-BisBAL NPs had increased tensile strength but diminished plasticity. However, these changes in mechanical properties interfered with neither their tissue adaption nor their application in slow-release topical delivery of the anti-tumor BisBAL NPs. Energy-dispersive X-ray spectra revealed nearly identical chemical compositions between the drug-free and Tecoflex™ EMs-BisBAL NPs except for the BisBAL NPs depositions in the Tecoflex™ EMs-BisBAL NPs. Small FTIR spectral differences were found between the Tecoflex™ EMs-BisBAL NPs and drug-free Tecoflex™ EMs, such as absorptions at 3449 and 3334 cm^−1^ in the NH region for Tecoflex™ EMs-BisBAL NPs, whereas drug-free Tecoflex™ displayed absorption at 3326 cm^−1^ due to free N-H in urethanes. It is hypothesized that NH groups that had been bound within the polyurethane became free NH moieties after interacting with the incorporated nanoparticles, although this displacement was not observed at the carbonyl region. Furthermore, the small shift in the CH_2_ bending that was observed at 1371 cm^−1^ was assigned to pristine BisBAL NPs, while the displacement and change in intensity of the C-N at 1247 cm^−1^ on Tecoflex™ EMs-BisBAL NPs was attributed to changes in surface phase separation.

Tecoflex™ EMs-BisBAL NPs had a hydration capacity of 94% after 24 h immersion. The water absorption of EMs is relevant for mediating their biological activities, both for management of exudative wounds and for the delivery of drugs contained within the membranes [35]. Thus, the water absorption capacity of Tecoflex™ EMs-BisBAL NPs is promising for local drug delivery. Regarding the mechanical properties, Tecoflex™ EMs have been reported to have good mechanical properties for biomedical application, even better than coronary arteries [36]. Moreover, BisBAL NP supplementation improved the resistance of Tecoflex™ EMs by 66%, although the plasticity was slightly reduced. For BC treatment where a low mechanical strength is favorable, this reduction in flexibility will not interfere with its performance as a delivery agent. The increased tensile strength of Tecoflex™ EMs due to the incorporation of BisBAL NPs will enable the topical delivery BisBALNPs in BC patients without Tecoflex™ EMs fraying.

In vitro studies have shown that drug-free Tecoflex™ EMs can stimulate the proliferation of fibroblasts, a characteristic not observed for dense Tecoflex^TM^ membranes that use arginine as a chain extender (PUUR) [15]. Other nanofibers, such as those based on polyethylene glycol and a Tecoflex^TM^ scaffold coated with an elastin-like peptide, also displayed excellent viability and proliferation in mouse fibroblasts and human vocal cord fibroblasts, respectively [37,38]. Furthermore, clear-field and fluorescence microscopy showed that healthy-looking HGF cells integrated well with drug-free Tecoflex™ EMs. Consistent with our findings, Venegas-Cervera et al., 2021 reported better elongation of human fibroblasts in EMs than in dense membranes [15]. Overall, our results support the biocompatibility of drug-free Tecoflex™ Ems [12], highlighting their potential for clinical application.

Tecoflex™ EMs-BisBAL NPs were bactericidal against both *MSSA*-ATCC and MRSA-CI, with a potency superior to vancomycin. The bactericidal capacity against methicillin-resistant *S. aureus* is noteworthy for potential clinical applications. The bactericidal activity of BisBAL NPs was higher than Tecoflex™ EMs-BisBAL NPs and this could be explained by the slow delivery of BisBAL NPs embedded into the electrospun membrane. Previously, our group described the antimicrobial and antibiofilm potential of BisBAL NP against different Gram-positive and Gram-negative bacteria, obtaining minimal inhibitory concentrations of 5 µM [20]. An EM loaded with 0.01 M Ag NPs also reduced *S. aureus* growth and biofilm formation after 6 h exposure [34]. A nanofiber membrane loaded with graphene oxide NPs decreased *E. coli* growth [39]. An electrospun PGLA/amorphous calcium phosphate bone nanocomposite reduced adhesion of clinical isolates of *S. aureus* and *S. epidermidis* [40]. In all, Tecoflex™ EMs-BisBAL NPs can be added to the list of promising bactericidal EMs.

Bismuth-based nanomaterials have been reported in use for several medical applications including treatment of different type of cancer [41]. A 24 h exposure of human BC MCF-7 cells to Tecoflex™ EMs-BisBAL NPs significantly reduced their growth by 87% (37 °C; 5% CO_2_). In contrast, drug-free Tecoflex™ EMs did not affect tumor cell growth. This result suggests that Tecoflex™ EMs can effectively deliver BisBAL NPs to the site of interest without harming healthy tissue.

Furthermore, BisBAL delivery by Tecoflex™ EM was highly efficient, reducing MCF-7 cell growth by 70% within 15 min. Although there has been a report on the antitumor efficacy of a biodegradable polycaprolactone EM loaded with paclitaxel in an in vivo xenograft nude mouse model [42], as far as we know, this is the first report on the efficiency of Tecoflex™ EMs loaded with an antitumor drug. Given the combined antibacterial and antitumor efficacy, Tecoflex™ EMs-BisBAL NPs could be applied immediately after mastectomy to prevent BC recurrence and postoperative wound infections. Given the absence of cytotoxicity of drug-free Tecoflex™ EMs, the material’s removal is not required while it continues to protect against BC recurrence. The insignificant cytotoxicity of bismuth nanomaterials has been observed in vitro and in vivo, with long-term biodistribution and renal excretion described in animal models [25]. BisBAL NP materials showed null cytotoxicity in a mouse model, where their antitumor efficacy was demonstrated [43].

The calcein AM assays further support the efficient antitumor activity of Tecoflex™ EMs-BisBAL NPs against the MCF-7 cell line, probably due to their affect on membrane permeability. The membrane-altering capacity of BisBAL NPs has been previously demonstrated by electron microscopy [21]. Other anti-tumor-specific mechanisms of action of BisBAL NPs include alteration of the cytoskeleton [44], apoptosis, and genotoxicity [21]. Thus, the action mechanism of BisBAL NPs differs from other nanoparticles, such as AgNPs, which cause loss of mitochondrial homeostasis and generate toxic reactive oxygen species [45]; or garlic–gold nanoparticles (As-GNPs), which mainly affect the microtubule scaffold in human breast adenocarcinoma cells (MDA-MB-231) [46].

## 5. Conclusions

Tecoflex™ EMs loaded with BisBAL NPs had antitumor activity against a breast cancer cell line and bactericidal activity against methicillin-susceptible and resistant *S. aureus*. The action mechanism is based on altered cell membrane permeability. Therefore, Tecoflex™ EM-BisBAL NPs may be useful as a topical treatment to prevent postoperative wound infections and BC recurrence after mastectomy.

## Figures and Tables

**Table 1:** FTIR spectra of drug-free EM and Tecoflex™ EMs-BisBAL NPs.

Chemical Bond	Absorption Wavelength (cm^−1^)	
	Drug-free Tecoflex™	Tecoflex™ EM-BisBAL NPs
Free N-H	3326	3449 and 3334
Asymmetric CH_2_	2932 and 2850	Similar
Symmetric CH_2_	2798	Similar
C=O (Amide I)	1716	Similar
N-H and C-H (amide II)	1525	Similar
CH_2_ bend	1446–1366	1371
C-N bend (amide III)	1228	1247
Polytetramethylene glycol C-O-C symmetric bend	1100	Similar
Urethane C-O-C symmetric bend	980	Similar
Tecoflex™ EMs-BisBAL NPs		997, 956, 864, 836
Cyclohexane symmetric bend	779	Similar

## Data Availability

The data are available from corresponding author after a request.
